# Dynamic characteristics of lipid metabolism in cultured granulosa cells from geese follicles at different developmental stages

**DOI:** 10.1042/BSR20192188

**Published:** 2019-12-23

**Authors:** Shanyan Gao, Xiang Gan, Hua He, Shenqiang Hu, Yan Deng, Xi Chen, Li Li, Jiwei Hu, Liang Li, Jiwen Wang

**Affiliations:** Farm Animal Genetic Resources Exploration and Innovation Key Laboratory of Sichuan Province, College of Animal Science and Technology, Sichuan Agricultural University, Chengdu, P.R. 611130, China

**Keywords:** Follicle development, Goose, Granulosa cell, Lipid metabolism

## Abstract

Previous studies have shown that lipid metabolism in granulosa cells (GCs) plays a vital role during mammalian ovarian follicular development. However, little research has been done on lipid metabolism in avian follicular GCs. The goal of the present study was to investigate the dynamic characteristics of lipid metabolism in GCs from geese pre-hierarchical (6–10 mm) and hierarchical (F4-F2 and F1) follicles during a 6-day period of *in vitro* culture. Oil red O staining showed that with the increasing incubation time, the amount of lipids accumulated in three cohorts of GCs increased gradually, reached the maxima after 96 h of culture, and then decreased. Moreover, the lipid content varied among these three cohorts, with the highest in F1 GCs. The qPCR results showed genes related to lipid synthesis and oxidation were highest expressed in pre-hierarchical GCs, while those related to lipid transport and deposition were highest expressed in hierarchical GCs. These results suggested that the amount of intracellular lipids in GCs increases with both the follicular diameter and culture time, which is accompanied by significant changes in expression of genes related to lipid metabolism. Therefore, it is postulated that the lipid accumulation capacity of geese GCs depends on the stage of follicle development and is finely regulated by the differential expression of genes related to lipid metabolism.

## Introduction

As the critical component of ovarian follicles, GCs play a pivotal role during follicular development and oocyte maturation [1–4]. Previous functional studies on GCs were mainly focused on proliferation, apoptosis and steroidogenesis [5–8]. Whereas, it has been recently reported that lipid metabolism in bovine, sheep and human GCs is also important for follicular development [9–11]. Investigations of lipid profile in both follicular cells (including cumulus, granulosa and theca cells) and follicular fluid by mass spectrometry (MS) suggest that lipid metabolism is pivotal for follicular development and oocyte maturation [12–16]. Additionally, glucose is known as the main energy source for the oocyte [17], since the oocyte has a limited capacity to utilize glucose and the substrates such as pyruvate and lactate are main energy sources metabolized by GCs [18,19]. Thus, lipid metabolism in GCs is considered to be indispensable for oocyte maturation.

By comparing the transcriptomic profiling of bovine cumulus cells (CCs, a type of GCs) isolated from ovaries before and after oocyte maturation, 27 genes related to lipogenesis, lipolysis, fatty acid transport and fatty acid oxidation (FAO) are identified [20], and 22 of them were significantly up-regulated from the phase of germinal vesicle breakdown to metaphase-II during *in vitro* maturation [20]. Furthermore, inhibitors of FAO (e.g. etomoxir) decreased the maturation rate of *in vitro* cultured oocytes in a dose-dependent manner by decreasing expression of lipogenic genes and increasing that of lipolytic and FAO-related genes [20,21]. Similarly, in bovine GCs, pharmacological inhibition of the enzymatic activity of fatty acid synthase using its specific inhibitors C75 significantly reduced the production of progesterone, suggesting that ovarian steroidogenesis relies on lipid metabolism in GCs [9,22]. In support of this, peroxisome proliferator activated receptor γ (PPARG), a key transcriptional factor regulating lipid metabolism, was widely expressed in rat, buffalo and sheep GCs, and was shown to participate in the synthesis of steroid hormones by modulating the aromatase activity [23–25]. These results suggested that lipid metabolism is important for follicular maturation by regulating cellular energy metabolism, proliferation, apoptosis and steroid hormone synthesis.

Changes in lipids have shown that the total lipid content increased with the growth of follicles (including early antral, small antral, medium antral, large antral, pre-ovulatory, ovulatory follicle) in goat ovary by biochemical quantitative [26]. Morphological observation also showed lipid droplet changed in shape from small and black to large and gray and its number increased in both oocyte and GCs as the follicles develop in mammals [27–29]. Our recently published study demonstrated that *de novo* lipogenesis (DNL) exists in goose ovarian follicles, especially in GCs [30]. Additionally, previous studies on avian GCs indicated that GCs change in their morphological and functional characteristics with the maturation of ovarian follicles. For instance, the number of GCs as well as their thickness and surface area change among avian follicles at different developmental stages [31,32] and the shape of GCs changes from flatten to cuboidal during follicle development [32]. Meanwhile, GCs are maintained in an undifferentiated state before being selected into the preovulatory hierarchy, and are active in mitosis but have weak steroidogenic ability [33,34]. And the pre-hierarchical GCs are more prone to undergo apoptosis than the hierarchical GCs [31,35]. Furthermore, the maturation of avian follicles is accompanied with the accumulation of yolk [36], and the major component of yolk is lipid. Taken together, we speculated that lipid metabolism in GCs is associated with the stage of follicle development in birds.

Therefore, the present study aimed to reveal differences in the dynamic characteristics of lipid deposition in the three cohorts of *in vitro* cultured GCs from geese follicles at different developmental stages and to investigate the expression profiles of a series of lipid metabolism-related genes in these GCs. These data are expected to lay a foundation for future research on follicular lipid metabolism and the underlying molecular mechanisms.

## Materials and methods

### Experimental animals

Tianfu meat geese, at the age of 35–45 weeks and laying in regular sequences of at least 2–3 eggs, were used in the present study. Geese were kept under natural conditions of temperature and light and had free access to food and water at the Experimental Farm of Waterfowl Breeding of Sichuan Agriculture University (Sichuan, China). Individual laying patterns were recorded on a daily basis. The healthy geese were killed by cervical dislocation 6–8 h ahead of oviposition. All procedures described herein were conducted according to the Guide of the Faculty Animal Care and Use Committee of Sichuan Agricultural University (Sichuan, China).

### Isolation and culture of GCs

Follicles were rapidly dissected from each ovary and were then washed with ice-cold phosphate-buffered saline (PBS, Solarbio Science & Technology Co., Ltd, Beijing, China). These follicles were classified into pre-hierarchical (<10 mm) and hierarchical (designated as F5-F1) follicles, and three cohorts of them including the pre-hierarchical (6–10 mm), F4-F2 and F1 follicles were further selected for isolation of GCs based on our previously protocol [35,37]. GCs isolated from each cohort were digested with 0.1% collagenase (sigma, Aldrich, U.S.A.), counted using the Handheld Automated Cell Counter (Millipore Corporation, Billerica, MA 01821). The GCs were seeded at a density of ∼3 × 10^5^ cells/well (12-well plate) and cultured as previously described [35], and these cells were used for Oil red O staining and qPCR.

### Oil red O staining and morphological observation

The amount of lipids accumulated in GCs was evaluated using the Oil red O staining method according to a previously described protocol with slight modification [38]. In brief, after being washed three times with PBS, cells were fixed with 4% paraformaldehyde (Solarbio Science & Technology Co., Ltd, Beijing, China) for 1 h. Then, the cells were washed twice with PBS, and were subsequently stained with Oil red O (Sigma, ST. Louis, United States) in the dark for 1 h at room temperature. Thereafter, cells were washed with PBS and photographed using a phase contrast microscope (Olympus, Tokyo, Japan). Finally, isopropanol was added into wells to extract Oil red O that transferred to 96-well plates and subjected to determination of optical density (OD) value at 490 nm using the automatic enzyme immunoassay analyzer.

### RNA extraction and cDNA synthesis

Total RNA was extracted from each sample using Trizol reagent (Invitrogen, Carlsbad, CA, U.S.A.), according to the manufacture’s instruction. The RNA quality, purity and concentration were using gel electrophoresis and spectrophotometric absorbance measurement. Equal amount of total RNA (1 μg) from each sample was reversely transcribed into cDNA using a cDNA synthesis kit following the manufacture’s instruction (TaKaRa, Shiga, Japan).

### Quantitative real-time PCR

The mRNA expression levels of those genes of interest were detected by quantitative real time PCR (qRT-PCR) using the SYBR PrimerScriptTM RT-PCR kit (TaKaRa, Shiga, Japan). The qRT-PCR reactions were conducted in a 12.5 μl volume (containing 1 μl cDNA, 6.25 μl of SYBR Premix^EX^ Taq, 4.25 μl of sterile water and 1 μl of primers) and were run on the CFX96TMReal-Time System (Bio-Rad, Hercules, CA, U.S.A.). The qRT-PCR procedures included 95°C for 10 s, followed by 40 cycles of 95°C for 5 s imposing the annealing temperature for 30 s and at 72°C for 10 min. An 80-cycle melting curve was performed, starting at a temperature of 65°C and increasing by 0.5°C every 10 s to determine primer specificity. Each sample was conducted in triplicate and the relative mRNA level for genes were normalized by β*-*actin and *18 s* rRNA using 2^−ΔΔ*C*^_t_ method [39]. Primer used for qRT-PCR are listed in Table 1.

**Table 1:** Primer pairs for real-time quantitative PCR

Genes	Forward primer (5′-3′)	Reverse primer (5′-3′)	*T*_m_ (°C)	Size (bp)
accα	TGCCTCCGAGAACCCTAA	AAGACCACTGCCACTCCA	60	163
fasn	TGGGAGTAACACTGATGGC	TCCAGGCTTGATACCACA	60	109
*dgat1*	CCTGAGGAACTTGGACACG	CAGGGACTGGTGGAACTCG	59	265
*dgat2*	CGCCATCATCATCGTGGT	CGTGCCGTAGAGCCAGTTT	59	113
*cpt1*	GTCTCCAAGGCTCCGACAA	GAAGACCCGAATGAAAGTA	56	193
*atgl*	TCGCAACCTCTACCGCCTCT	TCCGCACAAGCCTCCATAAGA	60	300
*apob*	CTCAAGCCAACGAAGAAG	AAGCAAGTCAAGGCAAAA	56	153
*mttp*	CCCGATGAAGGAGAGGAA	AAAATGTAACTGGCCTGAGT	56	85
*srebp1*	CGAGTACATCCGCTTCCTGC	TGAGGGACTTGCTCTTCTGC	60	92
*pparβ*	TGACGGCGAGCGAGAT	CAGGTAGGCGTTGTAGATGTG	60	83
*pparγ*	CCTCCTTCCCCACCCTATT	CTTGTCCCCACACACACGA	59	108
*β-actin*^1^	CAACGAGCGGTTCAGGTGT	TGGAGTTGAAGGTGGTCTCG	60	92
*18 S*^1^	TTGGTGGAGCGATTTGTC	ATCTCGGGTGGCTGAACG	60	129

1 Houskeeping gene for data normalization.

### Statistical analysis

Results were presented as the mean ± standard error of mean (SEM) of three independent experiments. All data were subjected to analysis of variance (ANOVA), and differences among the means were assessed for significance by Duncan’s multiple range test using SPSS (IBM, SPSS Inc., Chicago, IL, U.S.A.). *P* < 0.05 was considered statistically significant.

## Results

### Dynamic changes in morphological characteristics of lipid droplets among different cohorts of *in vitro* cultured GCs

**Figure 1:**
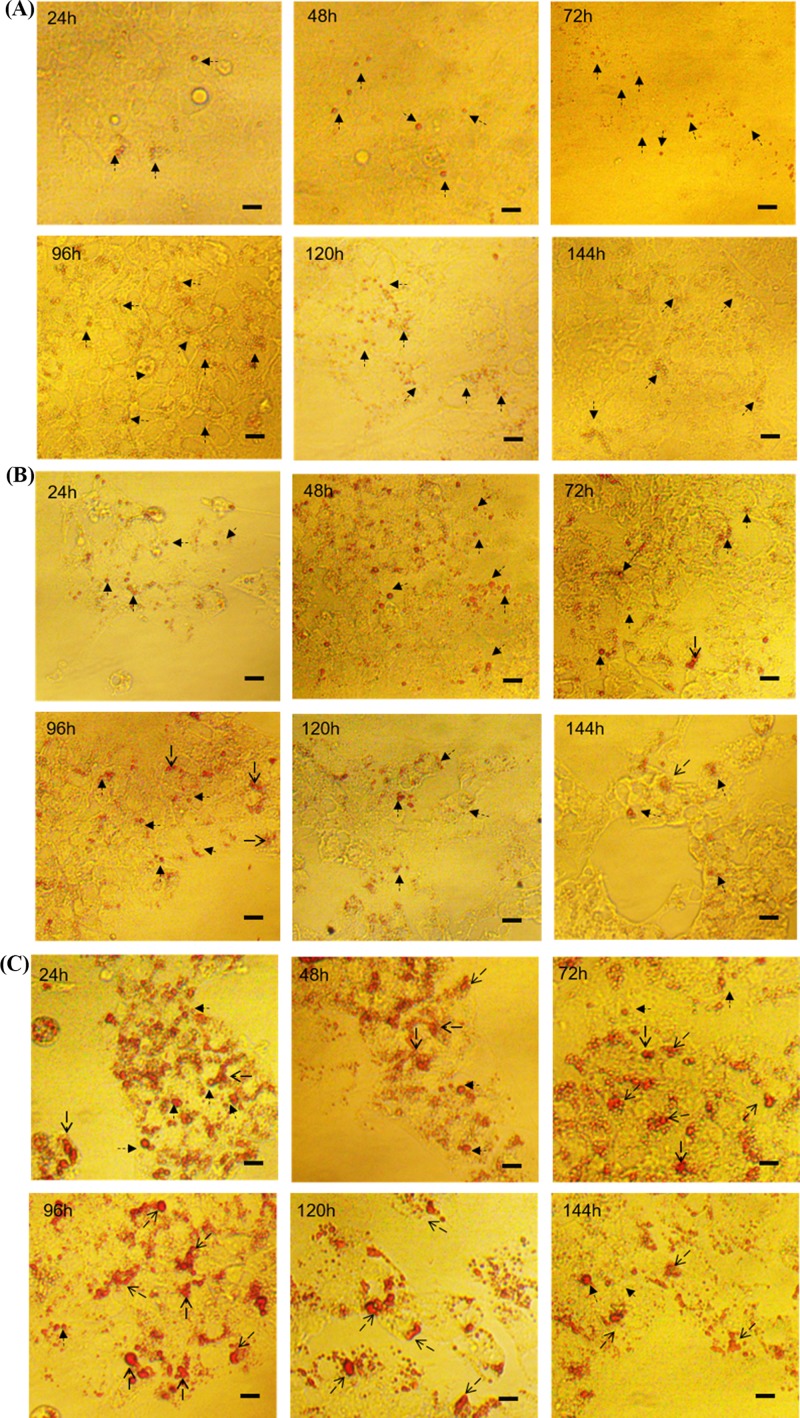
Morphological characteristics of lipid droplets *in vitro* cultured GCs Panels (**A–C**) represent the pre-hierarchical, F4-F2 and F1 GCs, respectively. The thin arrows represent round lipid droplets, and triangular arrows represent irregular lipid droplets owing to fusion. The scale marker represents 50 μm.

Changes in the morphology of lipid droplets among three cohorts of GCs isolated from follicles at different stages of development were evaluated using Oil red O staining and are shown in Figure 1. Morphological changes in lipid droplet were manifested by fusing with each other, and its size changed after fusion. With increasing incubation time, the pre-hierarchical GCs began to form smaller lipid droplets, which were generally spherical in shape. By contrast, both the F4-F2 and F1 GCs tended to form relatively larger lipid droplets, which were shown as spherical or irregular. Additionally, after Oil red O staining, the color of lipid droplets was generally brighter in the F1 GCs compared with the other two cohorts. Because the color intensity reflected the levels of triglycerides and cholesterol within lipid droplets, these data demonstrated that higher levels of lipids were accumulated in the hierarchical than pre-hierarchical GCs.

### Dynamic changes in the content of intracellular lipids among different cohorts of *in vitro* cultured GCs

**Figure 2:**
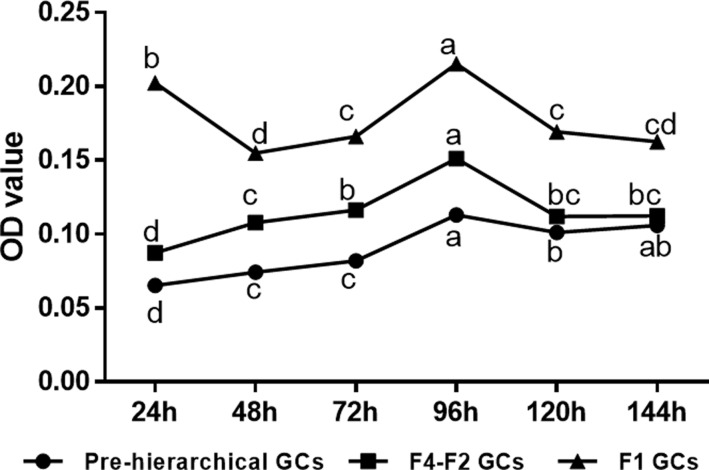
Intracellular lipid deposition of three cohorts of *in vitro* cultured GCs Different lowercase letters indicate the significant differences among different culture time within the same cohort of cells (*P* < 0.05).

Intracellular lipids within GCs were measured by Oil red O extraction. As shown in Figure 2. we found that large amounts of lipids were deposited into three cohorts of GCs after 24 h of *in vitro* culture. Furthermore, with the extension of incubation time, the amount of lipids accumulated in three cohorts of GCs increased gradually, reached the maxima at 96 h culture, and then decreased. Greater amount of intracellular lipids was found in hierarchical than pre-hierarchical GCs (*P* < 0.05) except the observation that during the incubation period from 120 to 144 h the F4-F2 and 6–10 mm GCs accumulated similar amount of lipids. Noticeably, an abrupt decline in intracellular lipids was seen in the F1 GCs at 48 h of culture. The results of Oil red O extraction indicated that a greater amount of lipids was shown to be accumulated in the F1 GCs compared with the other two cohorts throughout the incubation period, and the F4-F2 GCs appeared to have much higher ability than the 6–10 mm GCs in the accumulation of lipids.

### Expression profiles of lipid metabolism-related marker genes in cultured GCs of geese follicles at different developmental stages

**Figure 3:**
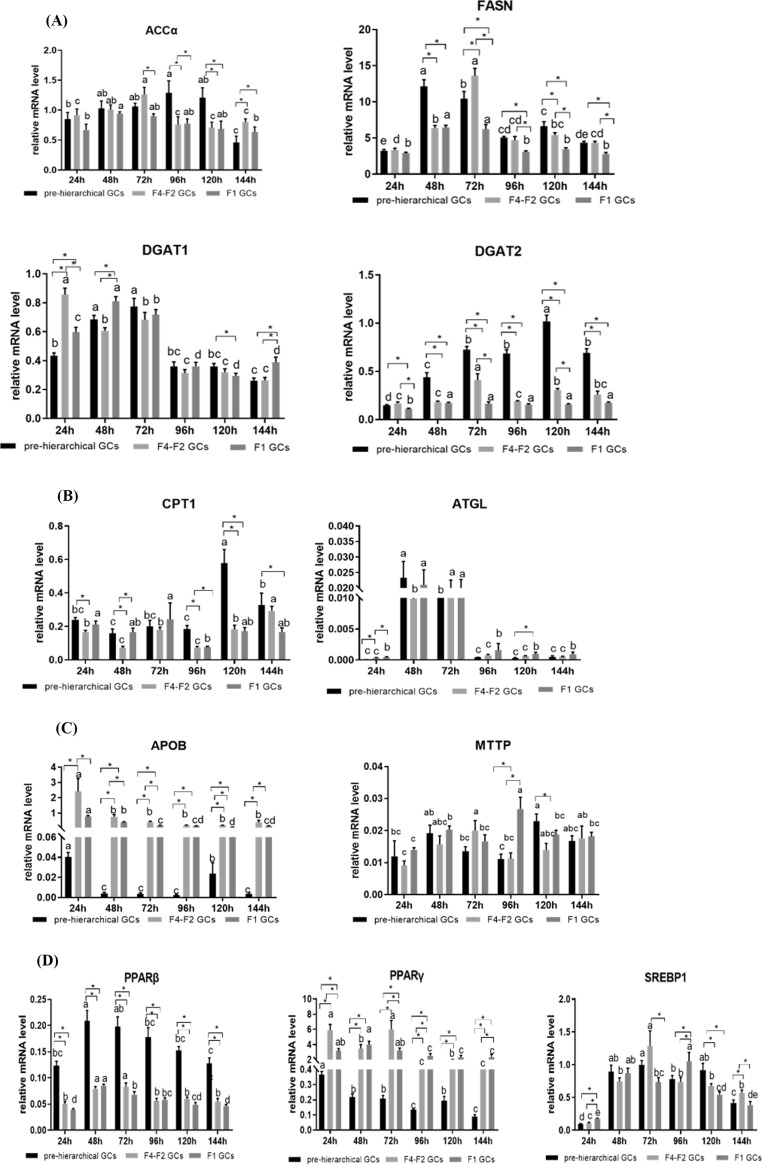
Expression patterns of genes involved in lipid metabolism *in vitro* culture GCs (**A**) Genes of lipogenic enzymes; (**B**) genes of lipolytic enzymes; (**C**) genes of lipid transport; (**D**) genes of transcription factors. Bars with different lowercase letters are significantly different for the same type of cell during the cultured time *in vitro* (*P* < 0.05). * indicates significant differences among GCs of three different stages in the same cultured time (*P* < 0.05). Data are presented as mean ± SEM (*n* = 3 replicate tanks). mRNA levels were normalized by *β-actin* and *18s*.

The mRNA levels of lipid metabolism-related marker genes were assessed by qRT-PCR. As shown in Figure 3A, lipogenic genes including acetyl-CoA carboxylase (*accα*), fatty acid synthase (*fasn*), diglyceride acyltransferase 1 (*dgat1*) and diglyceride acyltransferase 2 (*dgat2*) were expressed in all GCs but presented different expression patterns among three cohorts of GCs during *in vitro* culture period. While the mRNA levels of *accα* showed a significant increase in the pre-hierarchical GCs from 96 h to 120 h compared with hierarchical GCs (*P* < 0.05). The mRNA expression of *fasn* in the pre-hierarchical GCs was higher than that of the hierarchical GCs. The mRNA levels of *dgat1* showed a trend of increasing early and decreasing later throughout the incubation period. As for *dgat2*, its mRNA levels were higher in pre-hierarchical than in hierarchical GCs (*P* < 0.05), but there was no significant difference in its expression between the F1 and F4-F2 GCs (*P* > 0.05).

As shown in Figure 3B, carnitine palmitoyl transferase 1(*cpt1*), one of FAO-related genes, showed that a tendency of the levels of mRNA were higher in pre-hierarchical GCs than the hierarchical GCs. Adipose triglyceride lipase (*atgl*), lipolysis-related genes, the mRNA level was highest at 48 and 72 h. As for lipid transport, we measured the mRNA levels of apolipoprotein B(*apob*) and microsomal triglyceride transfer protein (*mttp*). Levels of *apob* were higher in hierarchical than in pre-hierarchical GCs (*P* > 0.05). The mRNA levels of *mttp* showed no significant changes. Additionally, we also measured the mRNA levels of transcriptional factors involved in the regulation of lipid metabolism including sterol-regulatory element binding proteins (SREBPs) and peroxisome proliferator-activated receptors (PPARs). Levels of *srebp1* increased with the increase of culture time, and then decreased (Figure 3D). The mRNA levels of *ppar*β had higher expression in pre-hierarchical GCs than hierarchical (*P* > 0.05). As for *pparγ*, the expression was highest in F4-F2 GCs.

## Discussion

In the present study, we primarily revealed the characteristics of lipid metabolism in cultured GCs from geese follicles at different developmental stages. During the entire incubation period, lipid deposition occurred in all three cohorts of GCs (Figure 1). With the extension of incubation time, the amount of lipids accumulated in these GCs increased gradually, reached the maxima after 96 h of culture, and then decreased (Figure 2). The morphology of lipid droplets (LDs) varied among these three cohorts, especially between pre- and hierarchical GCs. The pre-hierarchical GCs preferred to form smaller LDs that were generally spherical in shape. By contrast, both the F4-F2 and F1 GCs tended to form relatively larger LDs that were shown as spherical or irregular. Meanwhile, after Oil red O staining, the color of LDs was generally brighter in the F1 GCs compared with the other two cohorts (Figure 1). LDs are highly dynamic organelles, and they can store neutral lipids and are involved in many physiological processes [40–42]. The follicles of almost all mammals contain LDs, and LDs are considered to be a source of energy for oocyte maturation [43,44]. As ovarian follicles grow, it was reported that changes in both the size and number of LDs changed [43,45,46]. The total intracellular lipid content increased gradually with the follicular growth, and increased exponentially in large antral follicles [26,47]. Our study showed that the lipid content in GCs increased with the follicle development, which was similar to these reports. Since both secretion of steroid hormones and energy metabolism increased with the development of follicles, it was postulated that differences in the amount of lipids deposited in different cohort of GCs could be dependent on the stage of follicle development. And this may reflect the different capacities of the GCs to respond to hormonal signals and provide energy for follicle development. Our results showed that the lipid content of GCs increased with the development of goose follicles, and the morphology of LDs was related to the stage of follicular development.

**Figure 4:**
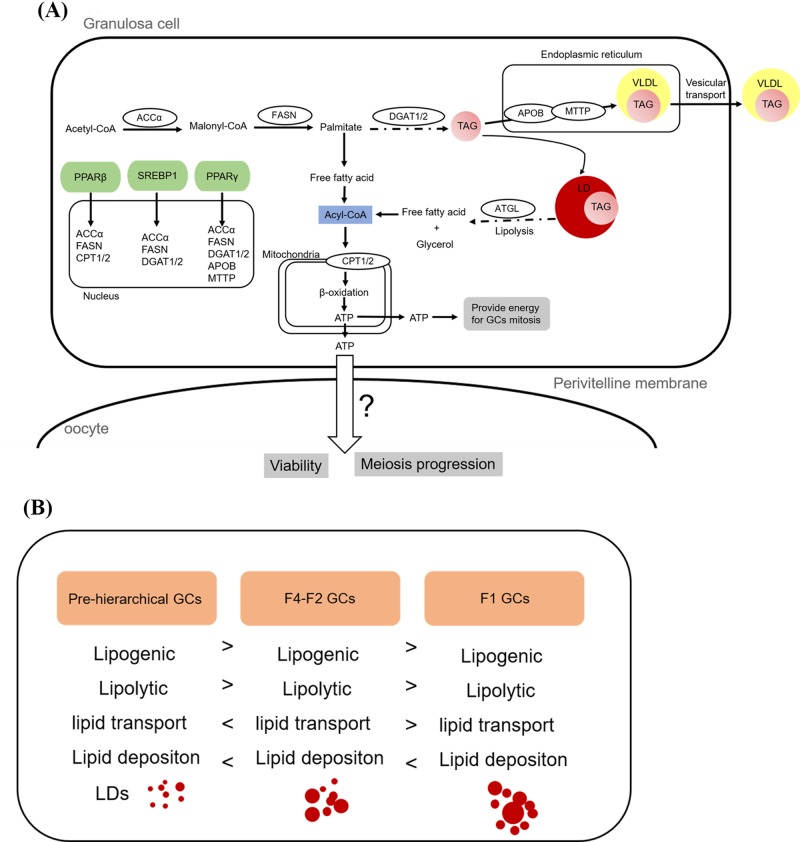
Schematic representation of the mechanisms possibly responsible for developmental stage-dependent changes in the capacity of lipid accumulation in GCs (**A**) Putative roles of lipid metabolism-related genes in goose GCs. (**B**) Differential expression profiles of genes related to lipogenesis, lipolysis, lipid transport, and lipid deposition lead to differences in the capacity of lipid accumulation in GCs of follicles at different developmental stages.

To further explore the lipid metabolism characteristics among the three cohorts of GCs, 11 genes associated with lipid metabolism were selected for qRT-PCR. Among them, *accα, fasn* and *dgat1*/2 were recognized as key enzymes in *de novo* synthesis of fatty acids and triglyceride synthesis, respectively [48–52]. Our results showed that the mRNA levels of *accα* and *fasn* were significantly higher in pre-hierarchical GCs than in the other two cohort cells in 96 and 120 h. And the mRNA levels of *dgat2* were also significantly higher in pre-hierarchical GCs than the other two cohort cells. At the same time, the mRNA expression pattern of *ppar*β was similar to that of *dgat2*. These results showed that pre-hierarchical GCs may synthesize more endogenous triglycerides than hierarchical GCs. The *apob* and *mttp* co-regulate of lipoprotein assembly and secret [53,54]. In our study, the mRNA levels of *apob* were higher in hierarchical GCs than pre-hierarchical GCs, and the mRNA levels of *mttp* showed no significantly changed among the three cohorts of GCs. *pparγ* is the lipid metabolism-related key transcriptional factor [55], and *pparγ* can regulate GCs steroidogenesis by influence aromatase activity [24] and loss of *pparγ* leads to reduce fertility [56]. The present study indicated that the mRNA levels of *pparγ* first increased and then decreased with follicular development that was consistent with previous studies [57,58]. Studies in mammals have shown that *dgat2* is related to the size of lipid droplets. When overexpressing *dgat2*, a large number of large lipid droplets are produced. [59]. Contrary to our study, in our study, the expression level of *dgat2* was higher in pre-hierarchical GCs than in hierarchical GCs, but the size of lipid droplets in hierarchical GCs was smaller than pre-hierarchical GCs. In addition, we know that phenotypic indicators are determined by multiple factors. Lipid deposition is determined by lipid synthesis rate, lipolysis rate and lipid transport rate. In our study, lipid deposition of hierarchical GCs is higher than that pre-hierarchical GCs. Why is this happening? It is not possible to explain the problem simply by quantification of several genes, but we can speculate that the expression of lipogenic genes in pre-hierarchical GCs is higher than in hierarchical GCs. But at the same time, the expression level of the lipolysis genes was also high (Figure 4A). Pre-hierarchical GCs mitosis are prevalent, so they require a lot of energy to supply their own metabolism. Put together, the lipid deposition of the pre-hierarchical GCs is less than the hierarchical GCs (Figure 4B).

In summary, these differences in expression of lipid metabolism-related genes and lipid content may be related to the stage of follicular development and reflect the function difference of GCs at different follicular development stages.

## Abbreviations

GC: granulosa cell
LD: lipid droplet
TAG: triglycerides
VLDL: very-low density lipoprotein
